# Comparative evaluation of the performance of the Abbott RealTime HIV-1 assay for measurement of HIV-1 plasma viral load on genetically diverse samples from Greece

**DOI:** 10.1186/1743-422X-8-10

**Published:** 2011-01-11

**Authors:** Antigoni Katsoulidou, Chrysoula Rokka, Catherine Issaris, Catherine Haida, Kimon Tzannis, Vana Sypsa, Maria Detsika, Dimitrios Paraskevis, Angelos Hatzakis

**Affiliations:** 1 National Retrovirus Reference Center, Department of Hygiene and Epidemiology and Medical Statistics, Athens University Medical School, Athens, 75 Mikras Asias Street, GR-115 27 Athens (Goudi), Greece

## Abstract

**Background:**

HIV-1 is characterized by increased genetic heterogeneity which tends to hinder the reliability of detection and accuracy of HIV-1 RNA quantitation assays.

**Methods:**

In this study, the Abbott RealTime HIV-1 (Abbott RealTime) assay was compared to the Roche Cobas TaqMan HIV-1 (Cobas TaqMan) and the Siemens Versant HIV-1 RNA 3.0 (bDNA 3.0) assays, using clinical samples of various viral load levels and subtypes from Greece, where the recent epidemiology of HIV-1 infection has been characterized by increasing genetic diversity and a marked increase in subtype A genetic strains among newly diagnosed infections.

**Results:**

A high correlation was observed between the quantitative results obtained by the Abbott RealTime and the Cobas TaqMan assays. Viral load values quantified by the Abbott RealTime were on average lower than those obtained by the Cobas TaqMan, with a mean (SD) difference of -0.206 (0.298) log_10 _copies/ml. The mean differences according to HIV-1 subtypes between the two techniques for samples of subtype A, B, and non-A/non-B were 0.089, -0.262, and -0.298 log_10 _copies/ml, respectively. Overall, differences were less than 0.5 log_10 _for 85% of the samples, and >1 log_10 _in only one subtype B sample. Similarly, Abbott RealTime and bDNA 3.0 assays yielded a very good correlation of quantitative results, whereas viral load values assessed by the Abbott RealTime were on average higher (mean (SD) difference: 0.160 (0.287) log_10 _copies/ml). The mean differences according to HIV-1 subtypes between the two techniques for subtype A, B and non-A/non-B samples were 0.438, 0.105 and 0.191 log_10 _copies/ml, respectively. Overall, the majority of samples (86%) differed by less than 0.5 log_10_, while none of the samples showed a deviation of more than 1.0 log_10_.

**Conclusions:**

In an area of changing HIV-1 subtype pattern, the Abbott RealTime assay showed a high correlation and good agreement of results when compared both to the Cobas TaqMan and bDNA 3.0 assays, for all HIV-1 subtypes tested. All three assays could determine viral load from samples of different HIV-1 subtypes adequately. However, assay variation should be taken into account when viral load monitoring of the same individual is assessed by different systems.

## Background

Quantitative measurement of plasma viral load provides a great insight into the pathogenesis of HIV-1 infection and constitutes an essential parameter of infection prognosis and optimal management of clinical patients [1-4]. Highly active antiretroviral therapy (HAART) results in a sharp decline of plasma HIV-1 RNA levels in patients and therefore initiation as well as treatment changes rely on correct determination of viral load levels [5,6]. Given the significance of accurate viral load measurements for optimal management of patients, the requirement for ultra-sensitive assays is crucial. A variety of commercial assays, are available for viral RNA levels quantification utilizing target or signal amplification technologies [7-12]. A major innovation of target amplification techniques is the development of assays that monitor accumulation of products in real-time, which are characterized by increased sensitivity, expanded dynamic range, diminished risk of contamination and high throughput [13,14].

HIV-1 shows a high level of genetic heterogeneity. Group M viruses are responsible for the majority of HIV-1 infections globally and according to the HIV nomenclature proposal, they are subdivided into nine subtypes (A-D, F-H, J and K), sub-subtypes (e.g. A1, A2, F1, F2), several circulating recombinant forms (CRFs) and unique recombinants http://hiv.lanl.gov[15,16]. In Greece increasing genetic diversity of HIV-1 has been documented and specifically an increase over time of the prevalence of non-B subtypes, particularly subtype A infections, has been reported [17].

Nucleic acid or signal amplification assays rely on the use of sequence specific primers and/or probes. HIV-1 increased heterogeneity may affect assay performance as the presence of natural polymorphisms in the target regions may reduce or inhibit hybridization thus compromising the reliability of viral load quantitation [18-20]. Virus subtype could therefore have a direct influence on assay performance.

The objective of this study was to compare the performance of the Abbott RealTime HIV-1 assay (referred to as the Abbott RealTime) (Abbott Laboratories, Wiesbaden, Germany) with the Cobas TaqMan HIV-1 Test (referred to as the Cobas TaqMan) (Roche Diagnostics GmbH, Manheim, Germany), and the Versant HIV-1 RNA 3.0 assay (referred to as the bDNA 3.0) (Siemens, Tarrytown, N.Y.) in order to determine the effect of viral heterogeneity on quantification of viral load.

## Methods

### Subjects and specimens

Whole blood was collected in sterile tubes with K_3 _EDTA as anticoagulant.

Within 4 h of drawing the blood, tubes were centrifuged at 1200× *g *for 12 min at room temperature. After centrifugation, aliquots of plasma were prepared and immediately frozen at -70°C until tested on the first thaw. This study was performed in compliance with regulations concerning human subject research and was approved by the Athens University Medical School Ethics Committee.

### Study design

The performance of the Abbott RealTime was compared to the performance of two pre-existing methods for viral load quantification routinely used in our laboratory. Two different sets of clinical samples from patients with established HIV-1 infection with or without antiviral therapy, previously assessed either by the Cobas TaqMan or the bDNA 3.0, were retrospectively chosen.

### Comparison of Abbott RealTime and Cobas TaqMan

A total of 149 plasma samples derived from HIV-1 positive patients of various viral load levels were retrospectively selected for testing with Abbott RealTime. Viral load levels of the same samples had been previously determined by the Cobas TaqMan by which 17 samples were found to have HIV-1 RNA values of <40 copies/ml, 21 samples had values ranging from 40 to 500 copies/ml and 111 samples had a viral load of >500 copies/ml (Table 1).

**Table 1:** Viral load values distribution of the samples used in the study.

	*Viral load (copies/ml)*	*N (%)*
	<40	17 (11.41)
	
***Samples selected according to***	40-500	21 (14.09)
	
***Cobas TaqMan result***	>500	111 (74.5)
	
	**Total**	**149**
	
	<50	12 (7.45)

***Samples selected according to***	50-500	32 (19.88)
	
***bDNA 3.0 result***	>500	117 (72.67)
	
	**Total**	**161**

### Comparison of Abbott RealTime and bDNA 3.0

A total of 161 plasma patient samples the viral load of which had been previously determined by the bDNA 3.0 were selected for testing with Abbott RealTime. bDNA HIV-1 RNA values were <50 copies/ml for 12 samples, 32 samples had viral load measurements ranging from 50 to 500 copies/ml and 117 samples were found with a viral load of >500 copies/ml (Table 1).

### Abbott RealTime assay

The assay uses reverse transcription polymerase chain reaction (RT-PCR) technology with homogeneous real-time fluorescent detection [21]. Sample preparation was performed manually according to the manufacturer's specifications (Sample Preparation System_DNA, _Promega Madison, WI, USA). RNA was extracted from 0.6 ml of plasma using magnetic microparticle technology and the HIV-1 RNA quantification range of the assay in the current protocol was 40-10,000,000 copies/ml. Isolated RNA was added manually to the prepared master mix, followed by real-time PCR amplification. Reverse transcription, PCR amplification, and detection/quantitation reactions, were performed on the Abbott *m2000rt *platform. Detection of PCR product in real time is based on a partially double-stranded probe which targets the integrase (IN) region of the polymerase *(pol) *gene [21]. The probe consists of two DNA fragments of different lengths: the longer fragment is complementary to the target DNA and is bound to a fluorescent marker, while the shorter fragment holds the quencher molecule. When the target DNA is not present, the long probe binds to the quencher probe and no fluorescence is detected; when the target DNA is present, the long probe preferentially binds to the target DNA and is able to fluoresce giving a quantifiable signal [21-23].

### Cobas TaqMan Test (for use with the High Pure System)

Cobas TaqMan is a competitive real-time RT-PCR based assay [24-26] and uses a dual labeled fluorogenic probe (TaqMan probe) targeting a highly conserved region of the HIV-1 *gag *gene. Sample preparation was performed manually using the High Pure System Viral Nucleic Acid Kit (Roche Diagnostics GmbH, Manheim, Germany) according to the manufacturer's specifications from 0.5 ml of plasma sample. The Cobas TaqMan 48 Analyzer was used for automated real-time RT-PCR and detection of PCR products [27-30]. Results calculations were performed based on parameters defined in the Test Definition File (TDF), in combination with AMPLILINK 3.0.1 software. HIV-1 RNA quantification range of the assay was 40-10,000,000 copies/ml according to the manufacturer's instructions.

### bDNA 3.0 assay

HIV-RNA was extracted manually from 1.0 ml of plasma sample. The bDNA 3.0 has a sandwich nucleic acid hybridization format and relies on signal amplification technology. Briefly, HIV-1 RNA is hybridized to a series of oligonucleotide probes complementary to highly conserved regions of the HIV-1 pol gene [7,31]. Hybridization and detection are carried out in a semiautomated system 340 bDNA analyzer (Siemens Medical Solutions Diagnostics, Tarrytown, NY), which automatically performs all incubations, washing steps, readings, and data processing. The assay has a dynamic range of 50 to 500,000 HIV-1 RNA copies/ml.

All three assays were carried out according to the manufacturer's instructions.

### Subtyping

HIV-1 subtype classification was performed on all samples, by DNA sequencing and phylogenetic analysis. Specifically, the sequences of protease (PR) and partial reverse transcriptase (RT) genes, used for HIV drug resistance routine testing, were determined either by the TrueGene HIV-1 Genotyping kit (Bayer Healthcare, LLC, Tarrytown, NY, USA) or the ViroSeq™ HIV-1 Genotyping System (Abbott Molecular Diagnostics, IL, USA). HIV-1 subtypes and recombinants were determined by phylogenetic analyses using a set of reference sequences including all previously described subtypes and circulating recombinant forms (CRFs) available from http://hiv-web.lanl.gov. Phylogenetic analysis of HIV-1 subtypes and recombinants was performed using Neighbor-joining (NJ) method with a HKY model of nucleotide substitution, as implemented in PAUP*4.0b10 [32]. Unclassified sequences were further examined for any evidence of recombination using bootscanning analysis, as implemented in Simplot 3.2 http://sray.med.som.jhmi.edu/SCRoftware/. Putative recombination pattern was further confirmed by phylogenetic analysis in each individual fragment with a distinct subtype assignment.

### Statistical analysis

Pearson's correlation coefficient was used to assess the strength of linear association between the log transformed values of Abbott RealTime and other methods. As the correlation coefficient provides information on the correlation but not on the agreement of the two methods, we further employed Deming regression [33] and Bland-Altman analysis [34,35]. Specifically, the fitted regression line, obtained using Deming regression for each comparison (Abbott RealTime versus Cobas TaqMan, and Abbott RealTime versus bDNA 3.0), was compared to the line of equality by testing the two-tailed hypothesis of slope = 1 and intercept = 0. Deming regression is similar to ordinary least-squares regression but it takes into account that viral load levels are measured with error by both methods. In the Bland-Altman analysis, the differences between the methods were plotted against their mean. The lack of agreement was then summarized by calculating the bias estimated by the mean difference $\overset{-}{d}$ and the standard deviation of the differences (SD). The limits of agreement were then estimated as $\overset{-}{d}\pm 1.96*\text{SD}$.

## Results

### Comparison of Abbott RealTime and Cobas TaqMan

The comparative performance of the Abbott RealTime and the Cobas TaqMan was assessed on 149 specimens. HIV-1 subtype information was available for 126 of them, with the following subtype distribution: 20 samples were characterized as subtype A, 90 as subtype B, seven samples as subtype C, one sample as subtype D, three samples as subtype F1, one sample as subtype G, two samples were found to be intersubtype recombinant strains A/B and two samples were characterized as CRF04_cpx Subtype determination was not possible for 23 samples either because of the low viral load or because of sample exhaustion (Table 2).

**Table 2:** Subtype distribution of the samples used in the study.

	Subtype HIV-1*	*N (%)*
	A	20 (13.42)
	
***Samples tested by Abbott***	Β	90 (60.40)
	
***RealTime and Cobas TaqMan***	Νοn A/Non B	16 (10.74)
	
	Unknown subtype**	23 (15.44)
	
	**Total**	**149**

	A	20 (12.42)
	
***Samples tested by Abbott***	Β	103 (63.98)
	
***RealTime and bDNA 3.0***	Νοn A/Non B	11 (6.83)
	
	Unknown subtype**	27 (16.77)
	
	**Total**	**161**

*HIV-1 subtypes were classified in three groups: subype A, B, non A/non B

** Subtype determination was not possible either because of low viral load or because of sample exhaustion.

Of the 149 samples 127 (85.2%) had a detectable viral load by both assays, 17 (11.4%) were undetectable by both assays and five (3.4%) samples were quantified only by Cobas TaqMan at 43.4, 50.8, 65.5, 257 and 843 copies/ml, respectively whereas the same five samples were reported as having HIV-1 RNA < 40 copies/ml by the Abbott RealTime. Thus, the detection rate of Abbott RealTime was 127/149 (85.2%) versus 132/149 (88.6%) of the Cobas TaqMan. Retesting of the discrepant samples by both assays revealed that only one sample characterized as subtype B (previously quantified at 843 copies/ml) was repeatedly reactive by Cobas TaqMan (674 copies/ml at retesting), while it was repeatedly non-reactive by Abbott RealTime. The remaining four samples were undetectable by both assays at following retesting (Table 3).

**Table 3:** Repeated testing of the samples with discordant results between Cobas TaqMan and Abbott RealTime.

Cobas TaqMan (+)/Abbott RealTime (-)	Repeat Cobas TaqMan	Repeat Abbott RealTime
(copies/ml)	(copies/ml)	(copies/ml)
43.4	<40	<40

50.8	<40	<40

65.5	<40	<40

257	<40	<40

843	674	<40

**Figure 1:**
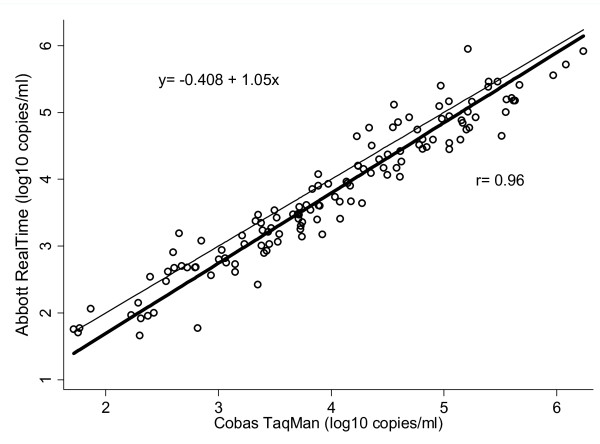
**Correlation between the Cobas TaqMan and the Abbott RealTime assays**. The bold line represents the regression line. The equation of the fitted line and the Pearson's correlation coefficient are presented on the plot. Values are expressed as log_10 _HIV-1 RNA copies/ml.

The two assays showed a high degree of correlation (Figure 1), and the linear regression equation was log_10 _(Abbott RealTime copies/ml) = -0.408 + 1.05 × log_10 _(Cobas TaqMan copies/ml), (correlation coefficient: r = 0.960, p < 0.001).

**Figure 2:**
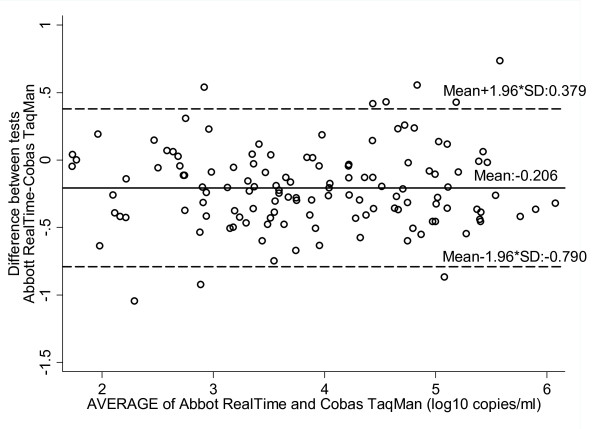
**Abbott RealTime and Cobas TaqMan degree of agreement (log_10 _copies/ml)**. The *x *axis of the Bland and Altman curves bears the mean values for each sample obtained by the two techniques. The *y *axis bears the differences between the values obtained by the two techniques. The solid line represents the mean difference between the values, and the dotted lines represent the mean difference plus or minus 1.96SD (95% limits of agreement).

Agreement between the two methods was calculated by the method of Bland Altman by plotting differences (log_10 _Abbott RealTime - log_10 _Cobas TaqMan) against the mean obtained by the two assays (Figure 2). On average, the Abbott RealTime gave values of 0.206 log_10 _copies/ml (SD:0.298, 95% limits of agreement, -0.790 to 0.379) lower than those obtained with the Cobas TaqMan assay. When differences were analyzed according to HIV-1 subtypes (classified in three groups) the mean differences (95% limits of agreement) between the two techniques for samples of subtype A, subtype B, subtype non-A/non-B were 0.089 (-0.602, 0.779), -0.262 (-0.739, 0.215), and -0.298 (-0.971, 0.375) log_10 _copies/ml, respectively.

Among the 127 patient samples in which viral load levels were determined by both assays, 108 samples (85%) differed less than 0.5 log_10 _copies/ml. A total of 18 samples (14.1%) differed from 0.5 log_10 _to 1.0 log_10 _copies/ml, with the following HIV-1 subtype distribution: two samples were of subtype A, 10 were found as subtype B, three as subtype C, two as subtype F1, and one as CRF04. Finally a viral load value difference between the Abbott RealTime and the Cobas TaqMan of >1.0 log_10 _copies/ml was observed for one subtype B sample (0.8%) with Abbott RealTime reporting the lowest value.

### Comparison of Abbott RealTime and bDNA 3.0

The comparative performance of the Abbott RealTime and the bDNA 3.0 was assessed on 161 specimens. HIV-1 subtype information was available for 134 of them, with the following subtype distribution: 20 samples were characterized as subtype A, 103 sample as subtype B, one sample as subtype C, three samples as subtype D, one sample as subtype F1, one sample as subtype G, one sample as subtype H, one sample was found to be an intersubtype recombinant strain A/B, two were CRF02_AG and one CRF04. HIV-1 subtype could not be determined for the remaining 27 samples, because of insufficient RNA levels or a lack of sample availability (Table 2).

Among the tested samples 142 (88.2%) had a detectable viral load by both assays, 10 (6.2%) were undetectable by both assays and nine (5.6%) showed discordant results. Specifically, discrepant results were observed for seven samples the viral load levels of which were reported at 54, 62, 66, 74, 76, 337 and 674 copies/ml by the bDNA 3.0. The same samples were found to have a viral load of <40 by the Abbott RealTime. Furthermore, two samples with viral load levels determined by the Abbott RealTime at 50 and 143 copies/ml, were reported to have a viral load of <50 copies/ml by the bDNA 3.0. The detection rate of Abbott RealTime, therefore, was 144/161 (89.4%) versus 149/161 (92.5%) of the bDNA 3.0. However, at the repeated testing of the seven samples detected only, six samples were reported to have viral load values of <50 copies/ml by the bDNA 3.0 and only one (previously quantified at 337 copies/ml) was quantified at 152 copies/ml (Table 4), whereas all were reported to have viral load levels of <40 copies/ml by Abbott RealTime. Finally, repeated testing of the two samples detected only by the Abbott RealTime was not possible because sufficient sample quantities were not available for further investigation.

**Table 4:** Repeated testing of the samples with discordant results between bDNA 3.0 and Abbott RealTime.*

bDNA 3.0 (+)/Abbott RealTime (-)	Repeat bDNA 3.0	Repeat Abbott RealTime
(copies/ml)	(copies/ml)	(copies/ml)
54	<50	<40

62	<50	<40

66	<50	<40

74	<50	<40

76	<50	<40

337	152	<40

674	<50	<40

*Repeated testing of the two samples detected only by the Abbott RealTime was not possible because of insufficient sample quantities

**Figure 3:**
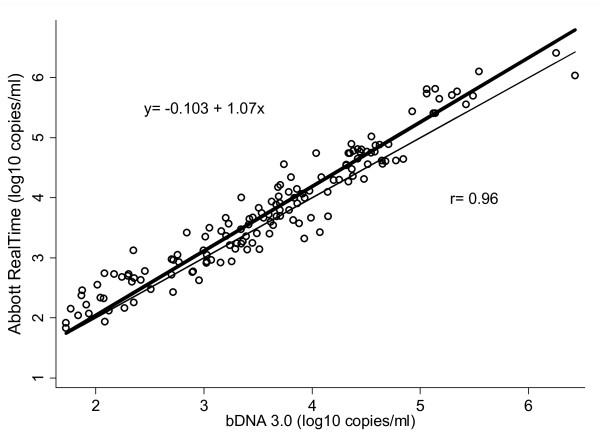
**Correlation between the bDNA 3.0 and the Abbott RealTime assays**. The bold line represents the regression line. The equation of the fitted line and the Pearson's correlation coefficient are presented on the plot. Values are expressed as log_10 _HIV-1 RNA copies/ml.

Figure 3 shows the scatter plot of log_10 _HIV-1 RNA copies/ml determined by the bDNA 3.0 and the Abbott RealTime assays, using specimens with detectable RNA levels by both assays. The fitted regression line was described by the equation: log_10 _(Abbott RealTime copies/ml) = -0.103 + 1.07 × log_10 _(bDNA 3.0 copies/ml), (correlation coefficient: r = 0.961, p < 0.001).

**Figure 4:**
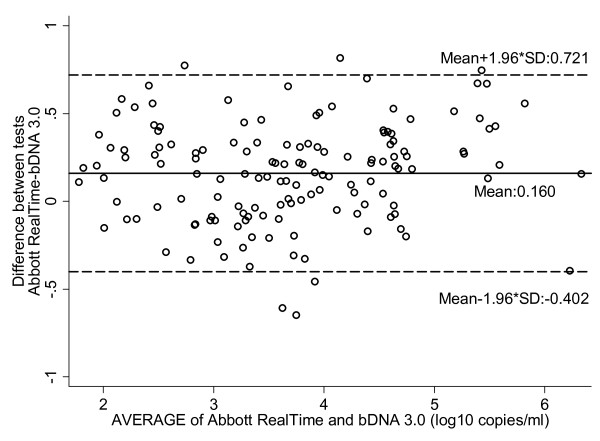
**Abbott RealTime and bDNA 3.0 degree of agreement (log_10 _copies/ml)**. The *x *axis of the Bland and Altman curves bears the mean values for each sample obtained by the two techniques. The *y *axis bears the differences between the values obtained by the two techniques. The solid line represents the mean difference between the values, and the dotted lines represent the mean difference plus or minus 1.96SD (95% limits of agreement).

Agreement between the two methods was calculated by the method of Bland Altman by plotting differences (log_10 _Abbott RealTime - log_10 _bDNA 3.0) against the mean obtained by the two assays (Figure 4). Viral loads values obtained by the two assays differed on average by 0.160 log_10 _copies/ml (SD:0,287, 95% limits of agreement:-0.402, 0.721). The mean differences (95% limits of agreement) between the two techniques for samples of subtype A, subtype B and subtype non-A/non-B were 0.438 (-0.097, 0.973), 0.105 (-0. 395, 0.604) and 0.191 (-0.418, 0.800), log_10 _copies/ml, respectively.

Among the 142 samples with a detectable viral load by both assays, 122 samples (86%) differed by less than 0.5 log_10 _copies/ml. Twenty samples (14%) differed from 0.5 log_10 _to 1.0 log_10 _copies/ml. HIV-1 subtype information was available for 17 of them, with the following subtype distribution: 9 A, 6 B, 1 CRF02_AG and 1 CRF04. Finally, none of the samples showed a deviation of more than 1.0 log_10 _copies/ml between assays.

## Discussion

Real-time RT-PCR assays for HIV-1 viral load measurements offer the potential of increased sensitivity, expanded dynamic range, diminished risk of contamination and high through-put. Given the importance of accurate viral load measurement for evaluating the efficacy of therapies and monitoring disease progression, and taking into account the vast genetic heterogeneity of HIV-1, it was our objective to compare the performance of the Abbott RealTime assay with the Cobas TaqMan and the bDNA 3.0 assays, using clinical samples from Greece. The recent epidemiology of HIV-1 infection in Greece has been characterized by increasing genetic diversity and a marked increase in subtype A genetic strains. Notably, subtype A infections have been found at similar frequency to subtype B among the recently infected individuals [17]. Due to the importance of HIV-1 RNA levels as a marker for HAART initiation and clinical management of HIV-infected individuals, the performance of the Abbott RealTime assay was questioned on a set of viral isolates including subtype A variants which have been shown to circulate in the form of a monophyletic clade in Greece. Since all these strains share a recent common ancestor, potential natural polymorphisms accumulating in regions targeted by Abbott RealTime could have caused discrepancies in the quantification of HIV-1 RNA that may also affect clinical practice.

Recently, Abbott RealTime with manual sample preparation was compared to the Roche Cobas Ampliprep/Amplicor HIV-1 Monitor v1.5 [36]. In the present study, Abbott RealTime with manual sample preparation was compared to two other ultra-sensitive assays, the Cobas TaqMan and the bDNA 3.0, in a routine setting of an AIDS reference laboratory, focusing on the inclusion of clinical samples of various viral load levels and various HIV-1 subtypes and CRFs. In all three assays the HIV-1 RNA extraction was performed manually, as that was the available protocol in Greece at the time.

A panel of 149 clinical samples previously tested with the Cobas TaqMan was also tested with the Abbott RealTime. Overall, the viral load results obtained with the two assays had a good correlation (r = 0.96). Correlation coefficients ranging from 0.79 to 0.96 have been reported previously [19,37-39].

All unquantifiable by Cobas TaqMan samples were also unquantifiable by Abbott RealTime. However, five samples previously quantified by Cobas TaqMan were undetectable by Abbott RealTime (Table 3). The possibility of contamination for these five samples was excluded. It should be noted that these five samples were selected according to their previous Cobas TaqMan result and three of them had marginally positive values (43.3 to 65.5 copies/ml). In order to determine whether the discordance between the two assays was reproducible, the five samples were retested by both assays, in which case all but one samples were found undetectable by both assays. One sample which previously quantified at 843 copies/ml was repeatedly reactive by Cobas TaqMan at 674 copies/ml (Table 3). The differences between the retesting values obtained by Cobas TaqMan could possibly be explained by the fact that the samples had a very low viral load. Measurements at such low levels are influenced by the inherent variability of assay technology, since the coefficient of variation for all these assays is greatest near the limits of their dynamic ranges. Indeed, increased variability in the lower viral load range (log2) seems to be typical for most viral load assays [22,23] and has been observed in previous comparative studies [26,31].

Other factors encountered that may have affected the reproducibility of HIV-1 RNA quantification include operator performance, test lot and the long-term storage of frozen plasma. In fact, because of the long period of time between assays we were unable to control for operator variability or test lot. Furthermore, although optimal storage conditions for HIV-1 RNA quantification have been followed [40], prolonged storage (12 months) of frozen plasma could possibly influence the values obtained at retesting especially in samples of such low viral load [40,41]. Interestingly, one subtype B sample repeatedly reactive by Cobas TaqMan at 843 copies/ml and 674 copies/ml could not be detected by Abbott RealTime at repeating the assay. This could possibly be explained by the presence of mismatches within primer/probe binding sites of the assay.

The viral load values assessed by Abbott RealTime in this study were on average lower (-0.206 log_10 _copies/ml) than those of Cobas TaqMan. This finding is in accordance with earlier studies where lower viral load values were also observed by the Abbott RealTime compared to the Cobas TaqMan [37-39,42], with the mean difference ranging from -0.34 to -0.20 log_10 _copies/ml. On the contrary, Gueudin et al., found an underestimation of viral load values by the Cobas TaqMan compared with the Abbott RealTime [19]. Moreover, in a recent study by Scott et al [23], a mean difference of 0.125 log_10 _copies/ml between the Abbott RealTime and the Cobas TaqMan has been reported in HIV-1 patients of subtype C. In the present study, differences between the two measurements exceeded 0.5 log_10 _copies/ml for 14% of the samples. Previously published studies reported differences of more than 0.5 log_10 _copies/ml (10.4% to 36.8%) in paired results between the two assays [19,23,38].

Interestingly, a more substantial difference (>1.0 log_10_) was observed for one subtype B sample for which lower viral load values were detected by the Abbott RealTime.

The reason for this misquantification is unclear and needs to be further investigated. One likely explanation for this difference in HIV-1 RNA quantification is the existence of mismatches within primer- or probe-binding sites that can have a significant impact on detection and accuracy of quantitation, although Abbott RealTime targets a highly conserved region of the HIV-1 genome, and reagents, cycling conditions, and probe design have been optimized for mismatch tolerance [21,22,43]. In earlier comparative studies between the two assays, viral load misquantifications of more than 1.0 log_10 _copies/ml have involved non-B subtypes (mainly CRF02_AG strains) with the Abbott RealTime generating higher viral load values compared to the Cobas TaqMan [19,23,44]. The genomic variability of HIV hinders the development of universal primers and probes for genomic hybridization. This holds for all HIV strains of different subtypes [45]. Most genomes available in sequence banks for primer and probe selection are mainly subtype B, with non-B strains representing a minority. Therefore, the risk of mismatches at primer and probe target sites is higher for non-B subtypes. However, even within subtype B, a simple synonymous mutation may reduce hybridization efficiency resulting in failed detection or inaccurate quantitation [19].

The Abbott RealTime was compared to bDNA 3.0 using 161 clinical plasma samples of various viral load levels and subtypes and a high correlation (r = 0.96) of the viral load results obtained by both assays was observed. Similar correlations of these two assays have been reported in the literature [22,46].

The Abbott RealTime could quantify two, undetected by bDNA 3.0, samples (viral load values 50 and 143 copies/ml). The assay could not be repeated for the two samples because of insufficient sample. Furthermore, a discrepancy between the two assays was initially observed for seven samples with detectable viral load levels by bDNA 3.0 (viral load values ranging from 55 to 674 copies/ml) and undetectable viral load levels by the Abbott RealTime. However, retesting of the samples by both assays, reported all but one samples undetectable by both assays (Table 4). The sample which previously quantified at 337 copies/ml was repeatedly reactive by bDNA 3.0 at a lower value of 152 copies/ml (Table 4). The possibility of contamination for these seven samples was excluded. As discussed previously, the differences between the retesting values obtained by bDNA 3.0 could be a result of very low viral load titers and prolonged sample storage. Finally, the sample which could not be detected by Abbott RealTime was of undetermined subtype. The presence of mismatches within primer/probe binding sites of the assay could possibly account for this discrepancy.

The viral load values assessed by the Abbott RealTime in this study were on average higher (0.160 log_10 _copies/ml) than those obtained by the bDNA 3.0, and this observation was consistent for all subtypes and in particular for subtype A. Between the two assays mean differences ranging from -0.14 to -0.01 log_10 _copies/ml have been reported previously [43,47,48]. In accordance with previous data [46], the majority of samples with a detectable viremia (86%) did not differ more than 0.5 log_10 _copies/ml among paired results. Notably, none of the samples differed more than 1.0 log_10 _copies/ml among the two assays.

Overall, the properties of the assays used for diagnostic purposes, such as the ones utilized in this study, in terms of how laborious, time consuming and cost effective they may be, vary. The overall turnaround time of the Abbott RealTime and Cobas TaqMan assays is significantly shorter than that of the bDNA 3.0 assay (5 h 15 min and ~24 h, respectively) as the latter involves a longer preparation process and a 16-18 hours incubation, whereas the two real-time assays require a two hour nucleic acid extraction step and a fully automated amplification and detection step. The assay sample throughput of Abbott RealTime as well as Cobas Taqman assays is 42 samples each in a full working day, whereas bDNA 3.0 assay has sample throughput of 84 samples per working day (plus the 16-18 h incubation). Although in this study the nucleic acid extraction step of Abbott RealTime and Cobas Taqman assays were performed manually, it is important to consider that both assays have options for increased testing scales with continuous sample loading on the automated extractor systems, which are not yet available in our lab. In that case, both assays can perform 96 samples/run with the m2000sp/m2000rt and COBAS Ampliprep/COBAS Taqman respectively. Furthermore, the two real-time assays require an initial volume of 0.5 and 0.6 ml, in contrast to the bDNA 3.0 assay that needs 1 ml, which may be a significant advantage where availability of sample volume is a matter.

## Conclusions

In this study, subtype-related variability in Abbott RealTime performance was evaluated by using samples from patients infected with HIV-1 subtype Α, Β and non-A/non-B (including circulating recombinant forms) strains. Specimens were quantified by Abbott RealTime with comparable viral load measurements to the other two assays. The viral loads obtained showed variations, with mean differences of -0.298 to 0.089 log_10 _copies/ml and 0.105 to 0.438 log_10 _copies/ml, depending on the subtype and the assay of choice. Thus, Abbott RealTime with manual extraction is an acceptable alternative to the conventional Cobas TaqMan and bDNA 3.0 assays for quantification of HIV-1 RNA in daily clinical routine and can be used for monitoring disease progression and the efficacy of antiretroviral therapy. Despite the strong correlation and good agreement observed between the Abbott RealTime and the other two assays, ongoing vigilance is recommended to evaluate assay performance with existing and emerging divergent strains. Furthermore, care is necessary when monitoring of viral load is performed with different assays, due to assay variability which may increase the risk of over- or underestimation of results. Finally, in accordance with relevant studies [44,49], in cases of discrepancy between viral load and CD4 count or clinical observations, measurement of plasma HIV-1 RNA with an alternative assay in order to highlight underestimation is reccmmended.

## Competing interests

The authors declare that they have no competing interests.

## Authors' contributions

AK participated to the study design and coordination and she prepared the manuscript. CR, CI and CH carried out the experiments. KT and VS performed the statistical analysis. MD participated in the editing of the manuscript. DP was in charge of the HIV subtyping project. AH was the study coordinator and participated in the writing and editing of the manuscript. All authors read and approved the final manuscript.
